# Renewable energy: A way out for South Sudan’s electricity crisis

**DOI:** 10.12688/f1000research.157192.1

**Published:** 2024-10-23

**Authors:** Aban Ayik, Nelson Ijumba

**Affiliations:** 1 African Centre of Excellence in Energy for Sustainable Development, College of Science and Technology, University of Rwanda, Kigali, Rwanda; 2 School of Engineering, University of Juba, Juba, South Sudan; 3 School of Engineering, University of KwaZulu, Natal, South Africa

**Keywords:** Electricity access, renewable energy, policy recommendations, strategic plan, South Sudan.

## Abstract

South Sudan is one of the least electrified countries in the world, despite having abundant renewable energy resources that could be exploited to generate electricity. The country relies on imported diesel for electricity generation, besides having limited focus on renewable energy development. This policy brief sheds light on the potential of renewable energy as a solution to South Sudan’s ongoing electricity crisis. It examines the key factors hindering the development of renewable energy resources for electricity generation in the country. The brief also provides recommendations to the Government of South Sudan, policymakers, experts, and funding institutions on how to improve electricity access in the country. It is stressing on the importance of prioritising the development of diverse renewable energy resources, such as solar, wind, and small hydropower, as an immediate solution to the electricity access challenges in the country.

## Introduction

In South Sudan only 5.4% of the population have access to electricity. ^1^ The country has no operational oil refineries and it depends on imported diesel for electricity generation. The current operational thermal power plants are mainly located in the capital city, Juba. In addition, there is no national electric grid to transmit the electricity generated in and around Juba to other parts of the country. The Government has been planning to improve the electricity access in South Sudan by building a national transmission network in addition to constructing a large hydropower plant. ^2^ However, most of these plans have not succeeded, mainly due to financial constraints.

A number of studies have shown that South Sudan has abundant renewable energy resources that can be exploited to generate electricity for almost the entire country. However, renewable electricity remains negligible in the power generation mix in South Sudan. Countries in the region such as Rwanda, Tanzania, Kenya, and Uganda are already improving their electricity access by using on-grid and off-grid renewable energy solutions such as solar, wind, and hydropower. So, what is delaying South Sudan from similarly unlocking its local renewable energy potential to improve its electricity access?

The aim of this policy brief is to examine the key factors delaying the development of renewable energy in South Sudan and to propose recommendations for improvement through exploitation of renewable energy resources.

## Policy outcomes and implications

**Figure 1.**
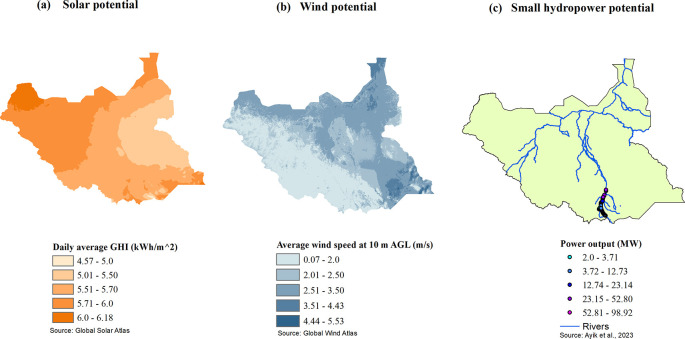
(a-c): Solar, wind, and small hydropower potential of South Sudan (sources: Global Solar and Wind Atlases and Ref. 4).

a) **Available renewable energy capacity**
Figure 1(a-c) shows the solar, wind and small hydropower potentials of South Sudan. Solar energy is the most abundant renewable resource throughout the country. Many locations receive annual global irradiation above 5.0 kWh/m ^2^, making it feasible for the development of large-scale solar power plants. Wind potential is generally low in the country, but there are areas where the annual average wind speed reaches 5.08 m/s at a height of 10 meters, ^3^ and therefore small wind turbines could be developed there. There are about 82 potential small hydropower sites along the White Nile and Kaya rivers, in the southern parts of South Sudan, which can produce a total output power of 165.69 MW. ^4^
b) **Factors limiting renewable energy uptake in South Sudan**
  The following were identified as contributing factors to the poor uptake of renewable energy in South Sudan:
  i. **Limited focus on renewable energy resources in South Sudan’s Strategic and National Development Plans**
    In South Sudan’s Vision 2040, renewable energy resources are not prioritised. Vision 2040 only highlights hydropower development as a strategic priority, with no mention of other renewable energy resources. In the Revised National Development Strategy (R-NDS) 2021-2024, ^2^ which is the current national development plan anticipated to guide South Sudan’s economic growth, it is stated that the country will invest in renewable energy, specifically hydropower. The Fula hydropower project was prioritised and allocated funding, and construction was scheduled to begin in 2022 and is expected to take 10 years to complete. However, work has not begun due to financial constraints.
  ii. **Reliance on imported electricity from neighbouring countries**
    Authorities in South Sudan have been focusing on importing electricity from neighbouring countries since 2011, although importing electricity is not a strategic priority in South Sudan’s national development plans. Electricity has been supplied to Renk, a county in the northern part of South Sudan, from Sudan since 2007 and continued after the country’s independence in 2011. ^5^ However, there has been a surplus of electricity because it could not be transmitted to nearby counties or towns due to the absence of a transmission grid. Thus, as of 2022, Renk was using only 5% of the imported electricity. ^6^ Additionally, since 2013, there have also been plans to import electricity from Ethiopia, which continue to this day. ^5, 7^ New plans include importing electricity from Uganda to provide power to towns at the border between Uganda and South Sudan.
c) **Implications**
  • **Lack of clear policy priorities**: Without clear goals and policy priorities for the development of renewable energy resources outlined in South Sudan’s long-term and medium-term plans, authorities may not be able to allocate resources or create necessary regulations and incentives to encourage their development.
  • **Unsustainability of electricity imports**: Importing electricity from neighbouring countries may not be sustainable because electricity can be interrupted by different geopolitical factors.
  • **Challenges with hydropower development**: Although hydropower is a renewable energy resource, it faces significant environmental challenges, in addition to schedule and cost overruns.

## Actionable recommendations

The following are actionable recommendations for the Government of South Sudan, policy makers, experts, and regional and international institutions that support development projects in the country:

i. **Prioritise the development of diverse renewable energy resources in future development plans for South Sudan**
  South Sudan has diverse renewable energy resources that can be used to generate electricity. While some locations lack large hydropower potential, they have abundant solar resources, along with potential for small wind turbines and small and mini hydropower development. Biomass and geothermal resources are also anticipated in some locations in the country. Therefore, policy makers should prioritise the exploration, assessment and development of other renewable energy resources beyond hydropower in the country’s future development plans.
ii. **Prioritise developing a phased plan to build a national transmission and distribution network**
  Building an electricity transmission network across a country, though costly, is essential for enabling increased access to electrical energy. Therefore, policy makers and experts should develop a comprehensive network construction plan that can be implemented in manageable phases. Regional and international financial institutions should prioritise funding such a project through grants and loans.
iii. **Invest in mini-grids and off-grid solar energy projects**
  Due to costs and physical terrain considerations, the national transmission grid would not cover the whole country. Therefore, there will still be a need for investing in mini-grid and off-grid systems, particularly in rural and remote areas, where the majority of the population lives. Since solar energy potential is high throughout South Sudan, solar energy-based mini-grids and off-grid systems can offer viable solutions to improve electricity access in these locations.
iv. **Incentivising investors and private energy property developers**
  To attract investors and private companies it is essential to make investments in renewable energy projects attractive. This is possible by providing grants and tax incentives, as well as simplifying regulations for investing in such projects.
v. **Conduct thorough techno-economic studies on importing electricity to South Sudan**
  It is imperative to conduct in-depth techno-economic studies to assess and compare the feasibility of importing electricity from neighbouring countries versus developing local renewable energy resources. Regional integration and cooperation through trade with neighbouring countries are beneficial and highly encouraged. Therefore, it is vital to thoroughly evaluate the technical feasibility, economic viability, and potential impacts and risks associated with such projects before their commencement to avoid future conflicts.

### Ethics and consent

Ethical approval and consent were not required.

## Data Availability

No data are associated with this article.
