# Renewable energy: A way out for South Sudan’s electricity crisis

**DOI:** 10.12688/f1000research.157192.3

**Published:** 2025-10-08

**Authors:** Aban Ayik, Nelson Ijumba

**Affiliations:** 1 African Centre of Excellence in Energy for Sustainable Development, College of Science and Technology, University of Rwanda, Kigali, Rwanda; 2 School of Engineering, University of Juba, Juba, South Sudan; 3 School of Engineering, University of KwaZulu, Natal, South Africa

**Keywords:** Electricity access, renewable energy, policy recommendations, strategic plan, South Sudan.

## Abstract

South Sudan is one of the least electrified countries in the world, despite having abundant renewable energy resources that could be exploited to generate electricity. The country relies on imported diesel for electricity generation, besides having limited focus on renewable energy development. This policy brief sheds light on the potential of renewable energy as a solution to South Sudan’s ongoing electricity crisis. It examines the key factors hindering the development of renewable energy resources for electricity generation in the country. The brief also provides recommendations to the Government of South Sudan, policymakers, experts, and funding institutions on how to improve electricity access in the country. It is stressing on the importance of prioritising the development of diverse renewable energy resources, such as solar, wind, and small hydropower, as an immediate solution to the electricity access challenges in the country.

## Introduction

South Sudan became independent in 2011 after a 21-year civil war that ended in 2005. From 2005 until independence, South Sudan was autonomous and organizations such as the World Bank and the United States Agency for International Development (USAID) were actively involved in development projects since that period. The country has also experienced internal conflicts after independence, particularly in 2013 and 2016. The civil war and internal conflicts contributed to damaging the country’s infrastructure, including its electricity infrastructure, and delayed various development projects. ^1^

South Sudan has a low population density, estimated at 13 people per km ^2^ in 2009, with more than 80% of the population living in rural areas, where they rely on fire wood for their energy needs. ^2^ This low population density has made it difficult to develop infrastructure and provide essential services efficiently, as the scattered population increases costs and reduces economic viability. ^2^

The electricity sector in South Sudan is underdeveloped. The exact number of people with access to reliable electricity is unknown due to a lack of official data. However, organizations such as the World Bank and IRENA estimated that in 2023, the percentage of the population with access to reliable electricity was between 5.4% and 8.4%. ^2^^,^^3^ Although South Sudan is rich in oil, it has no operational oil refineries and it depends on imported diesel for electricity generation. The current operational thermal power plants are mainly located in the capital city, Juba. In addition, there is no national electric grid to transmit the electricity generated in and around Juba to other parts of the country. The Government has been planning to improve the electricity access in South Sudan by building a national transmission network in addition to constructing a large hydropower plant. ^4^ However, government reports indicate that most of these plans have not succeeded, mainly due to financial constraints.

A number of studies have shown that South Sudan has abundant renewable energy resources that can be exploited to generate electricity for almost the entire country. ^5^^–^^7^ However, renewable electricity remains negligible in the power generation mix in South Sudan. Countries in the region such as Rwanda, Tanzania, Kenya, and Uganda are already improving their electricity access by using on-grid and off-grid renewable energy solutions such as solar, wind, and hydropower. So, what is preventing South Sudan from similarly unlocking its local renewable energy potential to improve its electricity access?

The aim of this policy brief is to examine the key factors delaying the development of renewable energy in South Sudan and to propose recommendations for improvement through exploitation of renewable energy resources.

## Policy outcomes and implications

**Figure 1.**
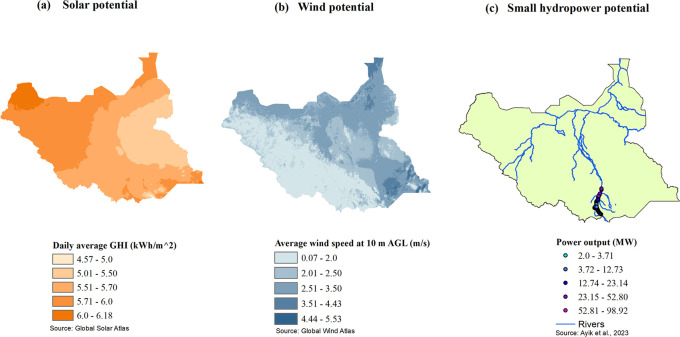
(a-c): Solar, wind, and small hydropower potential of South Sudan (sources: Global Solar and Wind Atlases and Ref. 9).

a) **Available renewable energy capacity**
Figure 1(a-c) shows the solar, wind and small hydropower potentials of South Sudan. Solar energy is the most abundant renewable resource throughout the country. Many locations receive annual global irradiation above 5.0 kWh/m ^2^, making it feasible for the development of large-scale solar power plants. Wind potential is generally low in the country, but there are areas where the annual average wind speed reaches 5.08 m/s at a height of 10 meters, ^8^ and therefore small wind turbines could be developed there. There are about 82 potential small hydropower sites along the White Nile and Kaya rivers, in the southern parts of South Sudan, which can produce a total output power of 165.69 MW. ^9^
b) **Factors limiting renewable energy uptake in South Sudan**
The following were identified as contributing factors to the poor uptake of renewable energy in South Sudan:
  i. **Limited focus on renewable energy resources in South Sudan’s Strategic and National Development Plans**
In South Sudan’s Vision 2040, renewable energy resources are not prioritised. Vision 2040 only highlights hydropower development as a strategic priority, with no mention of other renewable energy resources. In 2013, the World Bank recommended that South Sudan develop mini hydropower sites as a quick solution to improve electricity access while waiting for large hydropower projects, which are expected to take considerable time to be commissioned. ^10^
In the Revised National Development Strategy (R-NDS) 2021-2024, ^4^ which is the current national development plan anticipated to guide South Sudan’s economic growth, it is stated that the country will invest in renewable energy, specifically large hydropower. The Fula hydropower project (1080 MW) was prioritised and allocated funding, and construction was scheduled to begin in 2022 and is expected to take 10 years to complete. However, work has not yet begun due to financial constraints.
  ii. **Reliance on imported electricity from neighbouring countries**
Authorities in South Sudan have been focusing on importing electricity from neighbouring countries since 2011, although importing electricity is not a strategic priority in South Sudan’s national development plans. Electricity has been supplied to Renk, a county in the northern part of South Sudan, from Sudan since 2007 and this continued after the country’s independence in 2011. ^11^ According to official sources, South Sudan contracted for up to 40 MW of cross-border supply capacity from Sudan, transmitted mainly from the Roseires and Merowe dams to Renk. However, actual electricity consumption has been much lower and is measured in kWh/MWh at Renk substation annually by Sudanese teams for revenue calculations. ^11^ The imported electricity was expected to reach 140 MW by 2016 as it was anticipated to be transmitted to other towns nearby Malakal. However, the transmission line from Renk to Malakal has not been built and the distribution network has not been rehabilitated or extended as planned resulting in low demand. Thus, as of 2022, electricity used in Renk was estimated at only about 5% of the contracted supply capacity, reflecting very low capacity utilization. ^12^ Additionally, since 2013, there have also been plans to import electricity from Ethiopia, which continue to this day. ^10, 13^ New plans include importing electricity from Uganda to provide power to towns at the border between Uganda and South Sudan.
  iii. **Lack of an independent regulatory authority**
The South Sudan National Electricity Bill 2015 was developed to establish an autonomous electricity regulatory body to oversee “generation, transmission, bulk supply, distribution, supply, export, import, and operation of electricity, along with related matters” ^14^ in South Sudan. One of the main objectives of the Bill is the promotion of renewable energy in the country. However, up to date, the Bill remains inactive. Currently, the Southern Sudan Electricity Corporation Act, 2011, is the primary law governing the electricity sector in South Sudan. In the 2011 Act, there is no clear distinction between the roles of the government as operator, regulator, and policy maker. Also, the 2011 Act does not mention anything about supporting renewable energy in the country.
  iv. **Lack of private sector investment**
South Sudan Vision 2040 and the R-NDS recognize the role of the private sector in contributing to achieving economic growth and sustainable development in the country and the need for regulation to encourage their participation. Also, the 2015 Electricity Bill encourages private sector participation in the electricity market in South Sudan. However, there is lack of clear framework and incentives for private sector investment in renewable energy or electricity sector in the country.
  v. **Peace and stability**
South Sudan has a long history of war and internal conflicts both before and after independence. Since 2018, the country has been relatively peaceful, allowing for the implementing various projects, such as the rehabilitation and extension of Juba distribution lines, several road projects, and others.
Renewable energy projects generally have longer payback periods compared to thermal or diesel power plant projects. The payback period is the time required to recover the funds invested in the project. One of the most important factors in attracting investment in long term infrastructure projects, such as renewable energy projects, is ensuring that nothing interrupts the project implementation before it exceeds its payback period and starts generating profits. Therefore, investors need guarantees, through the political environment in the country, that South Sudan will remain peaceful and politically stable to encourage both local and foreign investment. The country must be stable, especially in locations anticipated for development.
c) **Implications**
  i. **Lack of clear policy priorities**: Without clear goals and policy priorities for the development of renewable energy resources outlined in South Sudan’s long-term and medium-term plans, authorities may not be able to allocate resources or create necessary regulations and incentives to encourage their development.
  ii. **Unsustainability of electricity imports**: Importing electricity from neighbouring countries may not be sustainable because electricity can be interrupted by different geopolitical factors. Additionally, importing electricity without proper planning can lead to underutilization of the contracted supply, which may result in higher costs.
  iii. **Challenges with large hydropower development**: Although hydropower is a renewable energy resource, it faces significant environmental and socioeconomic challenges. Hydropower contributes to aquatic life disruption as obstruction of river flow affects downstream habitats, machinery operation harms fish and other species, and imbalances arise between upstream and downstream ecosystems 1. ^3^ Besides, submerged vegetation and soil decompose, releasing methane and carbon dioxide. LHP projects can also experience schedule delays and cost overruns due to heavy construction, machinery installation, social conflicts and resettlement issues. ^15^
  iv. **Conflict of interest and poor safety standards:** Lack of a regulatory body can create an environment of lack of fairness and accountability, monopoly, corruption, conflict of interest, and poor safety standards. This can harm the public and the environment, discourage investors, and limit participation for investing in renewable energy projects.
  v. **Dependence on public funding and foreign loans:** As public funds alone are not enough to finance infrastructure projects, the lack of private sector investment may lead to limited investments and reliance on loans. As loans are paid back with interest, excessive loans can strain the national budget and create many economic problems which can increase the risk of renewable energy projects failure.
  vi. **Higher investment risks:** Lack of peace and stability in South Sudan may lead to higher investment risks which can discourage both local and international investors to invest in renewable energy projects.

## Actionable recommendations

The following are actionable recommendations for the Government of South Sudan, policy makers, experts, and regional and international institutions that support development projects in the country:

i. **Prioritise the development of diverse renewable energy resources in future development plans for South Sudan**
South Sudan has diverse renewable energy resources that can be used to generate electricity. While some locations lack large hydropower potential, they have abundant solar resources, along with potential for small wind turbines, biogas, and small and mini hydropower development. These technologies offer relatively lower capital cost compared to large hydropower projects. Also, some locations in the country have potential for the development of power from biomass and geothermal resources. Therefore, policy makers should prioritise, in the country’s future development plans, the exploration, assessment and development of other renewable energy resources beyond large hydropower in the country’s future development plans.
ii. **Prioritise developing a phased plan to build a national transmission and distribution network**
Building an electricity transmission network across a country, though costly, is essential for enabling increased access to electrical energy. Therefore, policy makers and experts should develop a comprehensive network construction plan that can be implemented in manageable phases. Regional and international financial institutions should prioritise funding such a project through grants and loans. Additionally, the government can look into partnership with the private sector.
iii. **Prioritise the establishment of an electricity regulatory authority**
To achieve sustainable energy goals, countries should establish institutions with clear policies and regulations to attract investments and grow their energy sectors, especially the renewable energy sector. ^16^ Therefore, South Sudan authorities and policy makers should prioritise the establishment of an autonomous South Sudan National Electricity Regulatory Authority. The authority is expected to be autonomous, while implementing government policies and regulations and managed by technical experts rather than by politically appointed individuals. This will ensure a clear separation of roles between the regulator and policymaker. It will also promote transparency and fairness, high safety standards, and encourage companies to invest in the electricity sector in the country, particularly the renewable energy sector.
iv. **Invest in mini-grids and off-grid solar energy projects**
It would not be possible for the national transmission grid to cover the whole country due to costs, low population density, and physical terrain considerations. Therefore, there would still be a need for investing in mini-grid and off-grid systems, particularly in rural and remote areas, where the majority of the population lives. Since solar energy potential is high throughout South Sudan, solar energy-based mini-grids and off-grid systems can offer viable solutions to improve electricity access in these locations.
v. **Incentivising investors and private energy property developers**
To attract investors and private companies it is essential to make investments in renewable energy projects attractive. This is possible by providing grants and tax incentives, as well as simplifying regulations for investing in such projects.
vi. **Conduct thorough techno-economic studies on importing electricity to South Sudan**
One of the benefits of the East African Community (EAC) regional integration is the sharing of energy resources through interconnections, where countries with excess electricity export to countries with lower capacity or higher demand. South Sudan can benefit from importing electricity from neighbouring countries to improve electricity access. However, it is important for South Sudan to first prioritize the development of its national transmission and distribution networks before planning to import electricity to ensure it can effectively transmit and distribute the imported electricity. Additionally, it is imperative to conduct in-depth techno-economic studies to assess the feasibility of importing electricity from neighbouring countries versus developing local renewable energy resources. It is also important to study the possibility of building transmission lines which support bidirectional power flow for potential future electricity trade with the region.

### Ethics and consent

Ethical approval and consent were not required.

## Data Availability

No data are associated with this article.
