# Supplementary material for: Renewable energy: A way out for South Sudan’s electricity crisis
Source: F1000Res. 2025 Oct 8;13:1267. Originally published 2024 Oct 23. [Version 3] doi: 10.12688/f1000research.157192.3 (PMC12511852; doi:10.12688/f1000research.157192.3)
Supplement: Peer review history

# Reviewer report: Reviewer response for version 3

**DOI:** 10.5256/f1000research.189284.r421735

**Published:** 2025-10-09

**Authors:** Taco Niet

**Affiliations:** 1 Simon Fraser University, Burnaby, British Columbia, Canada

Thank you for addressing most of my comments. I, however, still find the units and description in Factors ii confusing and unclear.

 Electricity imports are usually measured in MWh or GWh not MW.  Contracted capacity is different than capacity factor.  How often does the line get used at it's 40MW capacity?  If it is used sometimes at the 40 MW capacity, no matter how rarely, S. Sudan is getting the contracted capacity, irrespective of how much energy (MWh or GWh) is actually imported over a year on the line.

 "imported electricity was expected to reach 140 MW" again uses a capacity number for what should be an imported energy number (MWh).  How much energy is expected to be imported vs. what is actually imported (due to line restrictions, etc.)?

 "electricity used in Renk was estimated at only about 5% of the contracted supply capacity" again, electricity used (in GWh or MWh) doesn't correspond to 5% of a capacity figure (MW).

Does the paper provide a comprehensive overview of the policy and the context of its implementation in a way which is accessible to a general reader?

Partly

Is the discussion on the implications clearly and accurately presented and does it cite the current literature?

Yes

Are the recommendations made clear, balanced, and justified on the basis of the presented arguments?

Partly

Reviewer Expertise:

Energy Systems, Sustainable Energy, Energy Systems Modelling, Renewable Energy, Energy Storage

I confirm that I have read this submission and believe that I have an appropriate level of expertise to confirm that it is of an acceptable scientific standard.

# Reviewer report: Reviewer response for version 2

**DOI:** 10.5256/f1000research.179401.r374555

**Published:** 2025-05-29

**Authors:** Barnaby Joseph Dye

**Affiliations:** 1 King's College London, London, England, UK

I welcome the authors' attempts to engage in the reviews and make some substantial changes. I would still want further engagement with the politics of the subject matter and the policies discussed, and the dynamics of conflict which affect the ability to plan and deliver electricity in South Sudan, shaping the politics of what is generated, by what technology, where and delivered to whom. However, I understand that limits of what the authors are able to achieve here and the article provides a useful overview as well as technical details. 

 Policy Outcomes and Implications - Point V: 

 Is Political prioritization a factor/the ideology of those in power/ their priorities

             Also do they have ability to mobilize resources – do those in power control enough of the state and the country for a renewable energy policy to be enacted – is there enough political agreement across different parties. I wouldn’t expect this to be answered in depth, but a suggestion of these political factors would be welcome.

*Challenges with Hydropower Development - iii -* also environmental negatives and economic implications of that?

Does the paper provide a comprehensive overview of the policy and the context of its implementation in a way which is accessible to a general reader?

Partly

Is the discussion on the implications clearly and accurately presented and does it cite the current literature?

Yes

Are the recommendations made clear, balanced, and justified on the basis of the presented arguments?

Partly

Reviewer Expertise:

Development studies, Africa Studies, Political Economy, The politics of infrastructure and the electricity Sector in Africa

I confirm that I have read this submission and believe that I have an appropriate level of expertise to confirm that it is of an acceptable scientific standard.

# Reviewer report: Reviewer response for version 2

**DOI:** 10.5256/f1000research.179401.r374556

**Published:** 2025-04-02

**Authors:** Taco Niet

**Affiliations:** 1 Simon Fraser University, Burnaby, British Columbia, Canada

Overall you have addressed most of my points.  However, one point remains wrt the energy imported to South Sudan under Factors ii.

 1. 40 MW is not a suitable unit for imported electricity.  That may be the potential capacity but electricity imports are usually measured in MWh.

 2. It is unclear from the paragraph what the specific situation is.  Does South Sudan contract for up to 40 MW of capacity (continuously for the entire year) or for a given amount of electricity (in MWh)?  Specify what the contract says as these are very different things.

 3. The phrasing still says that South Sudan imports electricity that is then not used. **The electricity system does not work that way. ** It is possible that South Sudan pays for up to 40 MW of capacity but is unable to utilize that capacity due to constraints on the system, or does South Sudan contract and pay for up to 40 MWh of electricity each year but is unable to actually import/use it.  Clarify.

 Also, reference 13 does not link out correctly and goes to a 404 not found.  Please update the link.

Does the paper provide a comprehensive overview of the policy and the context of its implementation in a way which is accessible to a general reader?

Partly

Is the discussion on the implications clearly and accurately presented and does it cite the current literature?

Yes

Are the recommendations made clear, balanced, and justified on the basis of the presented arguments?

Partly

Reviewer Expertise:

Energy Systems, Sustainable Energy, Energy Systems Modelling, Renewable Energy, Energy Storage

I confirm that I have read this submission and believe that I have an appropriate level of expertise to confirm that it is of an acceptable scientific standard, however I have significant reservations, as outlined above.

# Reviewer report: Reviewer response for version 1

**DOI:** 10.5256/f1000research.172610.r347920

**Published:** 2025-02-04

**Authors:** Taco Niet

**Affiliations:** 1 Simon Fraser University, Burnaby, British Columbia, Canada

Summary:

 The article presents policy suggestions for South Sudan to expand electricity access.  The article argues that large hydro, imports and a dependence on oil are currently the focus in SS but that this should be revised to encourage deploying renewables across the country instead.  It also notes challenges building out the electricity grid due to financial constraints.

 There are a few issues that I think should be addressed:

 The context in SS is not well developed.  There was recent civil war that ended only a few years ago, and there is ongoing ethnic violence.  This makes it much more challenging to address energy issues and access capital for development of any sort.

 There is an implicit assumption that RE deployment will automatically increase electricity access.  This hasn't been established in the article and is not necessarily the case.  More imports and large power plants can also contribute to access.

 You discuss that importing electricity is not sustainable but also recommend continuing regional cooperation.  Reducing imports will likely reduce electricity access so hard to justify not continuing imports.  Also Recommendation v statements are counter to each other - encourage regional integration but also not.  Which is it?

 In Factors ii you say imported electricity is not used.  This is non-sensical - the grid doesn't work that way.  If electricity is imported it is used.  Clarify what you mean by this statement.

 Current electricity access (2022) is 8.4% https://data.worldbank.org/indicator/EG.ELC.ACCS.ZS?locations=SS.  But also 5.4% per IRENA's report.  Maybe give a range or discuss why the number might vary.  This is likely indicative of the issues in SS - lack of infrastructure makes it challenging to accurately assess such numbers.  Worth a mention.

 Recommendation i - might also discuss the fact that RE development requires smaller quanta of capital compared to large projects so can be easier to get going.

 Recommendation iv - Private equity does not show up in the policy issues so why is this a recommendation?  Where is the current policy issue with private equity?  This again speaks to the lack of context for SS as a country.

Does the paper provide a comprehensive overview of the policy and the context of its implementation in a way which is accessible to a general reader?

Partly

Is the discussion on the implications clearly and accurately presented and does it cite the current literature?

Yes

Are the recommendations made clear, balanced, and justified on the basis of the presented arguments?

Partly

Reviewer Expertise:

Energy Systems, Sustainable Energy, Energy Systems Modelling, Renewable Energy, Energy Storage

I confirm that I have read this submission and believe that I have an appropriate level of expertise to confirm that it is of an acceptable scientific standard, however I have significant reservations, as outlined above.

# Response

**Published:** 2025-03-13

**Authors:** Aban Ayik

**Affiliations:** African Centre of Excellence in Energy for Sustainable Development, College of Science and Technology, University of Rwanda, Kigali, Rwanda

- The context in SS is not well developed.  There was recent civil war that ended only a few years ago, and there is ongoing ethnic violence.  This makes it much more challenging to address energy issues and access capital for development of any sort.

*Response:* *As mentioned previously, the civil war in South Sudan ended 20 years ago. Despite the ongoing internal conflicts, the government has been able to access capital to implement some of its projects including energy projects. For context and background, we have added a brief paragraph providing an overview of the civil war and internal conflicts in South Sudan.*

- There is an implicit assumption that RE deployment will automatically increase electricity access.  This hasn't been established in the article and is not necessarily the case.  More imports and large power plants can also contribute to access.

*Response:* *RE deployment has increased electricity access in some countries in the region (Kenya, Uganda and Rwanda). Similarly, we expect it to do the same as studies indicate the availability of renewable energy sources in the country. However, the process is not automatic. Its success depends on the technical and economic feasibility, followed by proper implementation, supportive policies, and financing. Therefore, while large power plants and electricity imports can contribute to increased access, they will take longer to implement especially in the absence of a national grid. Instead, other options such as developing solar power plants, run-of-river small hydropower plants, mini hydropower plants, mini-grids or hybrid systems should be considered while larger projects are being developed. This manuscript has been updated to reflect this.*

  

- You discuss that importing electricity is not sustainable but also recommend continuing regional cooperation.  Reducing imports will likely reduce electricity access so hard to justify not continuing imports.  Also Recommendation v statements are counter to each other - encourage regional integration but also not.  Which is it? 

*Response: One of the benefits of the EAC regional integration is sharing of energy resources through interconnections, where countries with excess electricity export to countries with lower capacity or higher demand. So, we do not oppose regional cooperation or importing electricity because that can be beneficial in cases of high demand or for supplying remote border towns. It can also be beneficial if South Sudan plans to export electricity to the region in the future after developing its hydropower resources. However, we believe that prioritising the development of local energy resources will be more beneficial to South Sudan at the current time. South Sudan can prioritize developing its local energy resources while planning to import electricity.  This section of the manuscript has been revised for clarity.*

  

- In Factors ii you say imported electricity is not used.  This is non-sensical - the grid doesn't work that way.  If electricity is imported it is used.  Clarify what you mean by this statement.

*Response:* *South Sudan has been importing 40 MW of electricity (in bulk) from Sudan for use in Renk and Malakal towns, and the areas along Renk-Malakal road. However, the transmission line from Renk to Malakal has not been built and the distribution network has not been rehabilitated or extended, resulting in South Sudan paying for unused electricity. Due to low demand, Renk has been consuming only a fraction of the imported electricity. This section of the manuscript has been updated for clarity.*

  

-  Current electricity access (2022) is 8.4% https://data.worldbank.org/indicator/EG.ELC.ACCS.ZS?locations=SS.  But also 5.4% per IRENA's report.  Maybe give a range or discuss why the number might vary.  This is likely indicative of the issues in SS - lack of infrastructure makes it challenging to accurately assess such numbers.  Worth a mention. 

*Response: We agree. We have changed the access to a range between 5.4% and 8.4%.*

  

-  Recommendation I - might also discuss the fact that RE development requires smaller quanta of capital compared to large projects so can be easier to get going.

 
*Response: We agree.  This section has been updated.*

  

-  Recommendation iv - Private equity does not show up in the policy issues so why is this a recommendation?  Where is the current policy issue with private equity?  This again speaks to the lack of context for SS as a country.

 
*Response: We agree. This has been added.*

# Reviewer report: Reviewer response for version 1

**DOI:** 10.5256/f1000research.172610.r334853

**Published:** 2024-11-08

**Authors:** Barnaby Joseph Dye

**Affiliations:** 1 King's College London, London, England, UK

The construction of the problem – detail of what is possible and the negative implications of the present context are well made. The text is clearly articulated and the maps useful

- The word ‘Delay’ – assumes that South Sudan’s government wants to deploy renewables – or that it is inevitable
  - Are there interest groups set against?
  - The authors later mention that the government’s 2040 vision doesn’t support conventional renewables – only large hydropower
- The focus on large hydropower is rooted in longstanding ideas of development and electricity found across Africa, but also in Sudan (Which I presume South Sudan partly inherited?) and neighbouring  Ethiopia and Egypt
  - The authors are right to point to environmental problems and cost overruns and construction delays
- This seems to me a political problem therefore – simplistically a lack of ‘will’ – but which probably has deeper roots
  - I presume there are many invested in the oil economy and in importing refined oil from abroad? Are there also strong political interests in generators? In other countries like Nigeria these are key explainers of renewable non-development/lack of grid extension etc
  - At some stage this needs to be mentioned as a constraint or possibility for the future, or that understanding the political economy context would be a recommendation for investors/donors attempting to build new electricity infrastructure
- I am surprised that conflict and civil war is not mentioned once when it is so fundamental to the why infrastructure is difficult to achieve, constrained resources, lack of investment and the government’s ability to act. This is surely a key context even if beyond the scope of the recommendations here

Does the paper provide a comprehensive overview of the policy and the context of its implementation in a way which is accessible to a general reader?

Partly

Is the discussion on the implications clearly and accurately presented and does it cite the current literature?

Yes

Are the recommendations made clear, balanced, and justified on the basis of the presented arguments?

Partly

Reviewer Expertise:

Development studies, Africa Studies, Political Economy, The politics of infrastructure and the electricity Sector in Africa

I confirm that I have read this submission and believe that I have an appropriate level of expertise to confirm that it is of an acceptable scientific standard, however I have significant reservations, as outlined above.

# Response

**Published:** 2025-03-13

**Authors:** Aban Ayik

**Affiliations:** African Centre of Excellence in Energy for Sustainable Development, College of Science and Technology, University of Rwanda, Kigali, Rwanda

- The word ‘Delay’ – assumes that South Sudan’s government wants to deploy renewables – or that it is inevitable
-  We replaced the word “delaying” with “preventing”.
  - Are there interest groups set against?
  - No
  -  The authors later mention that the government’s 2040 vision doesn’t support conventional renewables – only large hydropower
  -  Yes, Vision 2040 supports hydropower (size not specified) but the government has mainly focused on large hydropower.

 

- The focus on large hydropower is rooted in longstanding ideas of development and electricity found across Africa, but also in Sudan (Which I presume South Sudan partly inherited?) and neighbouring Ethiopia and Egypt.
- Yes, the focus on large hydropower in South Sudan was indeed inherited from Sudan. In fact, almost all available environmental studies on hydropower projects in South Sudan were conducted before South Sudan’s independence.
  - The authors are right to point to environmental problems and cost overruns and construction delays
  - We appreciate the acknowledgment.

 

- This seems to me a political problem therefore – simplistically a lack of ‘will’ – but which probably has deeper roots
-  We agree. We also believe that the lack of a clear vision for the future development of electricity in the country is one of the main constraints.
  - I presume there are many invested in the oil economy and in importing refined oil from abroad? Are there also strong political interests in generators? In other countries like Nigeria these are key explainers of renewable non-development/lack of grid extension etc. 
  -  While diesel is also used for generating electricity, it is not the only reason for fuel imports in South Sudan. Imported fuel is also used for transportation (cars, buses, and tracks). Currently many government offices and some residents in Juba are using electricity from the Juba Electricity Distribution Company (JEDCO). So the demand for generators is not that strong as before.
-
  - At some stage this needs to be mentioned as a constraint or possibility for the future, or that understanding the political economy context would be a recommendation for investors/donors attempting to build new electricity infrastructure.
  - We agree. For example, there is no independent regulatory authority in South Sudan, and the government acts as a policymaker, regulator, and operator. This makes it difficult to maintain a stable and transparent environment for investment, which discourages investment in the sector.
  - A paragraph addressing this issue has been added to the manuscript.

 

- I am surprised that conflict and civil war is not mentioned once when it is so fundamental to the why infrastructure is difficult to achieve, constrained resources, lack of investment and the government’s ability to act. This is surely a key context even if beyond the scope of the recommendations here.
-  We agree that the civil war is a key context in the history of South Sudan, particularly in relation to damaged infrastructure. However, the civil war ended 20 years ago (in January 2005), leading to South Sudan becoming autonomous and later an independent country in 2011. There have also been internal conflicts (which are still ongoing) that have also contributed to further damage to the infrastructure. Despite these challenges, South Sudan has implemented various development projects since independence and during and after the internal conflicts, including the rehabilitation and extension of Juba distribution lines, several road projects, and others. So, while the civil war and internal conflicts had an impact, the current energy situation is mainly driven by other factors, such as ineffective planning, governance, and policy challenges. For context and background, we have added a brief paragraph providing an overview of the civil war and internal conflicts in South Sudan. We have also added political stability as a policy issue.
